# Challenges faced by community health volunteers in offering sexual and reproductive health care services to young women during the COVID-19 pandemic in Khwisero and Nairobi in Kenya

**DOI:** 10.3389/frph.2024.1491093

**Published:** 2025-01-07

**Authors:** Mariam F. Yusuf, Washington Onyango-Ouma, Jacinta Victoria S. Muinde, Cynthia Khamala Wangamati

**Affiliations:** ^1^ Institute of Anthropology, Gender and African Studies, University of Nairobi, Nairobi, Kenya; ^2^ Community Medicine and Global Health, University of Oslo, Oslo, Norway; ^3^ Sociology and Anthropology, Maseno University, Kisumu, Kenya

**Keywords:** sexual reproductive health services, community health volunteers, COVID-19, challenges, epidemics and pandemics

## Abstract

**Introduction:**

Community health volunteers (CHVs) are fundamental in many health systems across the globe. In Kenya, CHVs were essential in providing sexual and reproductive health (SRH) services during the COVID-19 pandemic. The study highlights challenges experienced by community health volunteers in Kenya while providing SRH services during the COVID-19 pandemic.

**Method:**

The study utilized a qualitative research design to explore challenges experienced by CHVs in offering SRH services in Dagoretti North and South sub-counties in Nairobi and Khwisero sub-county in western Kenya during the COVID-19 pandemic period. We conducted 17 in-depth interviews, 7 focus group discussions, and 1 group discussion with CHVs. The data was collected in different periods: in Khwisero, November 2022–August 2023, and in Nairobi, February–April 2023.

**Results:**

The study's findings show that CHVs experienced several challenges while providing SRH services. The challenges included distrust by the community as a result of the interaction between anti-COVID-19 measures and other past and present health interventions such as vaccines for other diseases, insufficient personal protective gear and equipment (PPEs), limited human and financial resources to address community needs, limited and poor training on response to misinformation that led to vaccine hesitancy, and mental strain from stigma and burnout.

**Conclusion:**

During pandemics, governments should provide adequate personal protective gear and financial and human resources for CHVs' work as they deliver SRH services to the community under risky conditions. In addition, CHVs must be trained to address misinformation about relevant medical interventions during epidemics and pandemics and provided with psychosocial support to mitigate the impact of psychological distress.

## Introduction

Sexual and reproductive health (SRH), related services, and rights remain a significant public health concern during different global or regional epidemics or outbreaks (1). During the COVID-19 pandemic, the pandemic and anti-epidemic measures were estimated to impact SRH care and outcomes negatively (2, 3). Extant research indicates that globally, COVID-19 negatively impacted access and utilization of SRH services (4) and the quality of the available services (1). During the pandemic, it was noted that access to SRH services embedded in SDG target 3.7, set for monitoring the progress towards achievement of SRH, such as contraceptive prevalence rate (CPR), adolescent sexual reproductive health, and safe abortion, was interrupted (5). Worldwide, SRH services were reported to be on the decline and, as a result, compromised and threatened the already achieved progress towards SRH set by sustainable development goals (SDGs), especially in developing regions in sub-Saharan Africa (SSA) (6). In many lower- and middle-income countries, such as those in SSA, access and utilization of SRH services was a challenge, and COVID-19 escalated the problem (3, 7).

Although governments in the SSA governments aggressively implemented strategies and measures to mitigate COVID-19 infection, the measures limited exposure to COVID-19 but caused broad disruptions to SRH services. Reports indicated that there was a decline in utilization of SRH services in the early months of COVID-19 due to fear of infection, long queues resulting from prioritization of COVID-19 services, limited and costly transport options, and additional costs such as out-of-pocket payments to purchase masks to access services (8, 9). During the first wave of the COVID-19 pandemic, sexual and reproductive health services in many SSA countries were either reduced or closed temporarily as they were considered unessential, and the limited financial and human resources were shifted to address COVID-19 (6).

In Kenya, the pandemic and resulting lockdown measures impacted SRH services since local resources were strained by long-term structural issues such as mobility, global and national supply chain disruptions, and de-prioritization of SRH services (10). Mobility restrictions affected both the community and the healthcare workers as movement and access to the healthcare facilities were restricted. Further, a report by the African Population and Health Research Center (APHRC) on SRH during the pandemic shows that most SRH commodities in Kenya experienced shortages linked to stockouts due to the Kenyan government “prioritization” of COVID-19 over other health services (11). This affected SRH services regarding cost and access because the availability of SRH supplies and commodities is critical to providing SRH services. For instance, although some health facilities in Kenya had commodities such as contraceptive injectables, these commodities were offered at an inflated cost due to high outsourcing costs from private entities (12).

Based on the limitations mentioned earlier, the WHO declared that SRH services were vital and essential and needed to be maintained throughout the pandemic (13). Following this directive, the Kenyan government, through the Ministry of Health, developed policies, guidelines, and regulations that enabled healthcare providers to continue delivering SRH services. In the early days of the pandemic, the government relied on non-pharmacological measures such as the mandatory wearing of face masks to slow down the transmission of the virus, hence preserving the health system's capacity to meet the much-needed SRH services (14). However, like in many parts of the world, Kenya remains a critical shortage of health workers (15). To address this gap, the Kenyan government required community health volunteers (CHVs) to provide different SRH services within their communities during the pandemic.

CHVs are a critical public health workforce as they work closely with the community, especially the economic and socially disadvantaged groups (16). According to Kahssay (17), they remain an integral part of many national health systems across the globe. During the global pandemic, in Kenya and elsewhere, CHVs were the contact people between health facilities and communities in offering sexual and reproductive health care services. For instance, CHVs continued accessing women in remote parts of different communities, extending family planning and reproductive health services (13).

However, despite recognizing CHVs' critical role in providing SRH services during the pandemic, they experienced challenges in delivering healthcare services. More research needs to be done in Kenya on the difficulties experienced by community health volunteers in providing SRH services. Therefore, this study aims to highlight challenges experienced by community health volunteers in Kenya while providing sexual and reproductive health care services during the COVID-19 pandemic.

## Methods

### Research design and study settings

The study employed a qualitative exploratory research design. The study is part of a more extensive study on COVID-19 and health systems and structures in Kenya. The study was conducted in two sites in Kenya: Nairobi and Khwisero. Nairobi is Kenya's capital city and largest urban area, with over 5 million occupants. Its urban footprint consists primarily of unplanned settlements driven by rapid population growth and sprawling informal settlements such as those in Dagoretti North and South (18). The growing population in Nairobi is attributed to in-migration (rural-urban), international entries, and high natural births. These factors conspired to expose Dagoretti North and South as one of the epicenter of COVID-19 infections in the initial stages of the pandemic in Kenya (19). On the other hand, Khwisero is one of the smallest sub-counties in Kakamega in Western Kenya. As a rural setting, Khwisero comprises 4 administrative units: Kisa Central, East, West, and North (20). CHVs in this region work in communities that are both socio-economically disadvantaged (20), and have high rates of SRH concerns such as cervical cancer, HIV/AIDS, low indicators of contraceptive uptake, low facility-assisted births, and poor antenatal visits (21–23). COVID-19 and contradicting information from within and elsewhere exacerbated the region's interaction with CHVs offering the different SRH services (24). Owing to that, the two sites were chosen because they showcase the urban-rural spatial linkages. The spatial linkages and connections between urban and rural in Kenya revolve around the flow and mobility of information, services, and people. However, during COVID-19, this was altered and tampered with as Nairobi was named the “hotbed” of the COVID-19 pandemic, with its occupants cautioned against traveling to the rural areas to mitigate the spread of the virus (25, 26). Mobility and transit choices were closely linked to the challenges experienced by CHVs in their SRH works in both regions.

### Study sample and sampling

The study participants were community health volunteers. In 2006, the government rolled out the Kenya Community Health Strategy to promote primary health care. The strategy mandates CHVs to deliver several health services to communities and link communities with the healthcare system (2, 3). In addition, CHVs offer health education, mobilize the community for health activities, refer patients to health facilities, and provide disease surveillance and reporting. Purposive sampling was used to recruit research participants. In the two sites (Nairobi and Khwisero), CHV participants working during the COVID-19 period were included in the study.

In Nairobi, we held interviews with 12 participants (3 males, 9 females) and focus group discussions (FGDs) with 35 participants (7 males, 28 females) (as shown in Table 1). In Khwisero, in-depth interviews were the lead method, triangulated with Group discussions (GD) and FGDs. We held repeated in-depth interviews with 6 participants (3 males, 3 Females), one group discussion with 14 participants (4 males, 10 females), and three Focus Group discussions with 30 participants (8 males, 22 females) (as illustrated in Table 1). Focus group discussions (FGDs) were held with a target number of participants segregated by gender, while the group discussion allowed for the expansion of the number of participants and inclusion of all genders, thus allowing for a discussion reflecting on different experiences across genders and ages in the same sitting. The group discussion took the shape of the local Khwisero community *barazas* (community meetings) that foster collaborative conversations (27).

**Table 1:** A description of the study participants.

Method	Age										Total	Place
	20–29		30–39		40–49		50–59		60–69			
	M	F	M	F	M	F	M	F	M	F		
IDIs	1	2			1	3	1	4			12	Nairobi
FGD1				2	1	4		1			8	Nairobi
FGD2			1	1	1	3		1		1	8	Nairobi
FGD3				4		2		3		1	10	Nairobi
FGD4			2	4	1	1			1		9	Nairobi
IDIs	2	1	1	2							6	Khwisero
GDs	2	6	2	4							14	Khwisero
FGD1	2		4		2						8	Khwisero
FGD2		6		4		1					11	Khwisero
FGD3		5		5		1					11	Khwisero

### Data collection

In Nairobi and Khwisero, the research assistants were trained on the qualitative data collection methods for three days, guided through the interview guides, and given guidelines on conducting IDIs and FGDs. Afterward, they were assigned two interviews to conduct and received feedback on areas for improvement before the commencement of the data collection process. The researchers listened to the interview recordings at the beginning of the data collection process and provided feedback to research assistants on how to probe more. Research assistants were also required to debrief the researchers at the end of the day about the data collection process and the challenges encountered. The researchers triangulated several data sources, e.g., in-depth interviews (IDIs) and focus group discussions (FGDs). The triangulation of data sources increased the richness of the data regarding challenges experienced as we gathered different perspectives from a large group of CHVs. We gained in-depth data from IDIs and multiple perspectives from groups of persons who were able to provide context to the opinions of the other members of the group (28).

In Khwisero, six repeated in-depth interviews (IDIs), three focus group discussions (FGDs), and one group discussion were conducted. All the interviews were audio recorded with consent from participants and transcribed. The first author checked the transcripts for accuracy. The interviews took place in the sub-county health facilities and private venues recommended by the research participants. Interviews were conducted in English and the local languages Swahili and Kisa, according to the participants' preferences. The GDs and FGDs took approximately 60–120 min, while the IDIs took approximately 45–60 min. Research assistants with multi-lingual experience in the local language (Swahili, English, and Kisa) were used to conduct the FGD and IDIs using interview guides.

In Nairobi, research assistants conducted 12 IDIs and 4 FGDs. A semi-structured interview guide was used to guide the IDIs and FGDs. The semi-structured interview guide was used because it allowed the researcher to have focused interviews with the freedom to explore arising topics during the interview, enhancing the understanding of the challenges faced by CHVs in providing SRH services during the COVID-19 pandemic (29). The IDIs and FGDs were audio recorded with consent from research participants. The audio-recorded interviews took place in private spaces recommended by the research participants. In-depth interviews and focus group discussions were conducted in English and Swahili, depending on the participant's preferred language. The interviews and group discussions took approximately 30–60 min and 60–120 min, respectively. The interviews were later transcribed verbatim. The fourth author checked for the accuracy of transcripts.

### Data analysis

Thematic inductive data analysis was applied by identifying, analyzing, and reporting patterns within the data (30). The iterative inductive data analysis involved (a) data familiarization, (b) initial codes generation, (c) search for themes, (d) reviewing themes, and (e) defining and naming themes (30). The transcripts and field notes were read several times to gain an in-depth understanding of the data. The transcribed data was imported into and coded using NVIVO 14 by the first and fourth authors. First, the first and fourth author independently coded their data and then met to discuss their codes and sub-themes as they handled different topics related to COVID-19 during their data collection process with CHVs. The first author focused on COVID-19 and SRH, whereas the fourth researched maternal health services and COVID-19. Afterward, they met on Zoom five times to discuss the sub-themes, with feedback from the third author. The sub-themes were similar, with a few differences highlighted in the results section. The first and fourth authors then developed a table of subthemes, which were reviewed, deliberated, and agreed on by all the authors. The first and fourth authors are trained in qualitative research and have extensive experience with thematic analysis.

### Ethical considerations

The study was approved by the National Commission of Science, Technology, and Innovation Kenya (NACOSTI) and Maseno University Scientific and Ethics Review Committee (MUERC/01063/22). All study participants were informed about the study's aim, purpose, and benefits, and participation was voluntary, as participants could withdraw from the study at any time without consequences. Informed written consent was obtained from study participants, and the research principles as per the Helsinki Declaration were observed.

## Results

Several themes emerged from the study. They included: (i) Communities' fear of contracting COVID-19 from CHVs, (ii) CHVs' psychological distress, (iii) limited resources, (iv) use of own resources, (v) training on Covid-19 vaccines, its side effects, (vi) vaccine hesitancy, and (vii) distrust of the community health volunteers.

### Communities' fear of contracting COVID-19 from CHVs

COVID-19 severely impacted Nairobi County. Since CHVs in Nairobi were part of the team that was handling COVID-19 patients, community members, including pregnant women, mothers, and women of reproductive age, feared contracting COVID-19 from them and did not welcome them in their households. A CHV stated:

I can say that during that time, we lost people within the households I oversaw. So, that really created some panic and fear about COVID-19. You will find that as a CHV, as much as you would go into the community wearing all the necessary protective gear, people would still fear you because they would associate you with the caregiving role and relationship that you had with the deceased. Community members were really scared of us because they felt that we were not scared [of COVID-19] or felt threatened about us visiting households that had people infected with COVID-19. (R4-FGD-CHV-Kawangware).

### Mental distress

Community health volunteers experienced mental distress from several sources. In Khwisero, the psychological impact on CHVs working on SRH issues during the pandemic was an important topic discussed.

None of the women we thought we had bonded with wanted anything to do with us; some had labeled us as COVID-19 virus carriers due to our work…and some started spreading rumors that we were sterilizing women by giving them the vaccine. All these narratives really took a toll on me and other CHVs. It was not only that the narratives were untrue, but also the isolation and stigma had affected our own families. (P1-IDI-CHV- Khwisero).

Notably, in Khwisero, some CHVs were forced to quit because of the increased workload, emotional exhaustion, and a stressful environment. The distress, social isolation, and burnout forced some CHVs to quit and leave the work.

I had a close CHV friend of mine. We have worked together in this community health work for long. She just one day decided to quit. She could not handle the stress and burnout that came with the work. She oversaw the maternal healthcare community services and complained that the community had created a hostile environment due to the CHV's lack of proper information and equipment to understand or educate them on their fears. She just left. (P3-IDI-CHV- Khwisero)

### Limited resources

Community health volunteers were not provided enough personal protective equipment (PPEs) during the COVID-19 pandemic. In the early period of COVID-19, there was a general lack of PPEs globally. However, more PPEs were manufactured and made available over the pandemic period. Community health volunteers complained that they had few PPEs, putting them at risk of infections. Community health volunteers elaborated:

A CHV does not have gloves. At times, you find a woman giving birth in the house, and you do not have gloves to render help, yet the woman might be HIV-Positive. Sometimes, as a CHV, you do not have the money to buy a razor blade for cutting the cord. There are no antiseptics, not even Dettol. There are no tools for CHVs to work with. (R3-CHV-FGD-Kawangware)

The challenge was further exacerbated by a lack of transport facilitation, corruption, and favoritism, where resources were distributed unfairly. Community health volunteers explained:

“You know our work during the pandemic was not easy. Sometimes, we had to walk very long to reach every household. We would go without food and transport money. But that was not even the worst part; our health was also constantly at risk due to the office not giving us enough masks and hand sanitizers. Some health workers will take the masks and sanitizers and keep them at their houses or give their friends, forgetting that we were the soldiers on the ground and needed to be properly equipped.” (P6-FGD-CHV- Khwisero)

“I would not say that the resources were not there. There were enough resources for everyone. However, our seniors at the health center decided to distribute the resources unfairly. One time, I was complaining to my fellow CHVs about the masks, transport, and sanitizers, and she wondered why I was complaining, yet she had all the things I was mentioning. When I inquired how she got it, she answered, “You should know people”. How am I supposed to know people when this is not my own personal work but the work of the state, which requires fair treatment for all health workers involved? To tell you the truth, I was demoralized.” (P2-IDI, CHV-Khwisero)

### Use of own resources

Community health volunteers visited households that were struggling economically as many people lost jobs during the COVID-19 period. When visiting households, they were forced to use their own resources to support vulnerable households, as explained in the excerpts below:

A challenge that we face in households is that you have been visiting a pregnant woman, but they have never planned on the resources they will require. For example, you may find a woman in labor but has no fare to take her to the hospital, yet you have been teaching them about birth plans. Therefore, because I am known to them, they call me to look for means by which they will get to the hospital. That forces me to use my money to support them. (R3-CHV-FGD-Waithaka)

Due to COVID-19 infection control and prevention measures, many people lost jobs, leaving households vulnerable. Most of the families had little to survive on, so CHVs were forced to buy them food from their own resources during household visits. A CHV proposed a kitty during the pandemic to cater to the needy. The CHV said:

Most of these houses have many challenges such as hunger. You might find a pregnant woman has stayed hungry. Therefore, we should at least have a kit to help us support them. After we have identified households with pregnant women, it is good that when we visit them, we at least have a kit to buy them something because it is for such reasons that we go deep in our pockets. When you visit a pregnant woman, and she tells you that she is hungry, you cannot leave her in that state. You buy her something, even if it is just milk. So, at times, we utilize the stipend in that manner. (R1-CHV-IDI-Kawangware)

When CHVs visited the households, there was an expectation for them to provide food and non-food items to communities. This is because many households lost their source of income, and communities expected governmental support regarding food and non-food items. When CHVs, seen as government representatives, failed to provide food and non-food items to the community, they were refused entry into homes, and communities were not receptive to their health messages. A CHV explained:

Many women did not want to come to the clinic or bring their children during that period. Even while we were going to persuade them to come to the health facility and bring their children to the clinic, they used to dismiss us, saying we were telling them about COVID-19 all the time instead of maybe bringing them food. Issues around accessing food brought many problems for pregnant women and kids. We were even afraid of paying them visits. (R7-CHV-FGD-Kawangware)

…. [during COVID-19] it was difficult to go into the households because they [community members] would refuse us to access their homes empty-handed…. (R6-CHV-FGD-Mutuini)

### Training on COVID-19 vaccines

Amongst the study participants in Khwisero, CHVs felt that the training on COVID-19 from the National level to the county to the sub-county level was done poorly. The training did not teach them about vaccines and their safety and impact on maternal and reproductive health. There were many rumors about the COVID-19 vaccines and their impact on reproductive health, yet these concerns were not addressed during the trainings. Community health volunteers said:

“We expected proper training on every aspect of the vaccine- the pandemic and how we were to navigate reproductive and maternal health care concerns during the pandemic, but everything was done in a rush. We, too, took the vaccine without detailed knowledge, so how could we convince people of something we had no proper knowledge of.” (P1-GD-CHV-Khwisero)

“There was a lot of information going around in the community and on social media about the vaccine and how they get mixed up in reproductive and maternal health, but we had no time to be trained on how to navigate all these issues. It really affected our interaction with the community- I still carry with me the emotional violence meted on me through words of insults by the villagers. But I do not blame them; everyone was and still is confused.” (P10-FGD-CHV-Khwisero)

The CHV work in Khwisero was hindered by a lack of proper training on COVID-19 and COVID-19 vaccines and the different ways to navigate misinformation concerns.

### COVID-19 vaccine hesitancy

During the intense COVID-19 period, there was much misinformation about vaccination. There were rumors that COVID-19 vaccines had negative effects on the reproductive health of pregnant women, mothers, and women of reproductive age. People talked of vaccines causing miscarriages, infertility, and reducing milk production for breastfeeding mothers. These rumors developed hesitancy in mothers towards the COVID-19 vaccine. In some homes, husbands restricted their wives from attending reproductive health care services because of rumors that women were being forced to be vaccinated when attending these services. Community health volunteer explained:

…. Most people were refusing to be vaccinated, especially pregnant women, because they would say that should they get vaccinated, they would miscarry. Those with kids [breastfeeding mothers] would say that should they get vaccinated, breast milk would dry up. We tried encouraging them against those assumptions so that they could accept and come for the vaccine. (R5-CHV-FGD- Mutuini, Nairobi)

“In male-headed households where the men were against the vaccines, they forced their women to stay home and not attend any antenatal or reproductive health care service at the hospital for fear of them being vaccinated. If you went to such a household to offer referrals or information on ANC or post-natal services or even vaccines, you would receive insults or be physically dealt with. One man slapped me when he found me in his house.” (P2-IDI-CHV-Khwisero)

The community resistance and vaccine hesitancy exposed CHVs to violence by community members.

Further, vaccine hesitancy also affected the CHV referral work on the different SRH services at the community health centers. Most health facilities had integrated COVID-19 vaccines into different services, such as maternal and child health services and antenatal care. Consequently, communities resisted any referral given by the CHVs to the health facilities.

The women I used to visit and refer to the different maternal or sexual reproductive health clinics no longer wanted my services or my presence. They accused me of referring them to the facilities so that they could inject them with a vaccine that would render them infertile… no one was ready to take in the vaccine and thus avoid other SRH services (P6-IDI-CHV- Khwisero)

### Distrust of the community health volunteers

During the early period of COVID-19, part of the prevention and mitigation measures involved quarantine of infected individuals. CHVs were tasked with tracing infected persons and sometimes assisting in taking people to quarantine centers. Many people resisted going to quarantine centers and, as a result, were forcefully removed from their homes and transported to quarantine centers. Following these activities, CHVs were feared in the community and hence affected their relationship with women of reproductive age, pregnant women, and mothers. A CHV explained:

I heard of two cases. One was pregnant, but she did not want it to be known. I made a follow-up on the two cases and ensured that they came to the facility for the BCG and check-up. It was because of the fear that they did not want us [CHVs] to know. They branded us as people in charge of COVID-19 who would take people for isolation. They never wanted us to know about anything. There was a barrier between us and them; we used not to interact the way we were used to; they would keep quiet whenever they saw us or isolated us. (R10-CHV-FGD-Mutuini)

Before the COVID-19 pandemic, other vaccination exercises were ongoing in the communities. Of interest to us is the HPV vaccine in schools in Khwisero, Kakamega. The vaccination process was rolled out through the Ministry of Health. Without consulting parents, vaccinations were forced on some children who reported to their parents and created tensions between the community and CHVs: CHVs mobilized schools and helped health workers with the vaccination process. A CHV said:

“I remember in one of the vaccination drives in Matarachi (pseudonym) primary school, we, the CHVs, were tasked to hold still the children resisting vaccination by attempting to escape. One of the children reported the scene to the parent who had yet to be informed of the vaccination drives happening in her child’s school- the parent had my number; I mean, this is a small village. She called me and insulted me for 20 min and rendered all sorts of threats. She even promised to tell others not to be part of any health services we would take to the community. I knew I was not at fault; I only followed instructions, so I told her to take it up with the government.” (P8-FGD-CHV- Khwisero)

On different occasions, CHVs were tasked by the government and different organizations to undertake registration exercises to enroll communities in interventions such as the universal health coverage project by the state, food donations, or financial support to households. Some of the organizations made promises that were not fulfilled, for example financial support. Consequently, this created mistrust in CHVs and affected their work. A CHV explained:

So, as CHVs we normally help various organizations do registrations for various activities. The challenge comes in when these organizations do not fulfill whatever they are registering people for. When that happens, people will still come to you saying that you registered them for something, yet you were not involved. At that time, people took advantage of the community. They would come and do something that does not sit well with community members and therefore destroy the name of the CHV, yet they might not be people or an organization that you had partnered with, but it will still come back to you as a CHV. (R8-CHV-FGD-Kawangware)

There was a time when they sent us to register pregnant women and nursing mothers. They signed forms, and we were told that they would have their hospital bills paid. We took all their details, and then a whole year passed without them having their hospital bills paid. It is very challenging to go to the same person to register them, so they resist when they are asked to give their information. (R2-CHV-FGD-Waithaka)

COVID-19 exacerbated tensions between CHVs and the community, increasing distrust and hindering their work during the pandemic.

## Discussion

This study, using qualitative data, highlights the numerous challenges faced by CHVS in offering SRH to women in Khwisero and Nairobi, Kenya, during the COVID-19 pandemic period. The study also provides recommendations for policymakers responsible for responding to and mitigating pandemics' impacts.

The close contact of CHVs with community members creates close relationships bound by trust. Vanden Bossche et al. (16) confirms that the trustful relationship with the community underpins the role of SRH service delivery by CHVs. Further, CHVs work attributes and contribute to establishing community bonds and relationships with broader health systems, especially on matters related to SRH that are personal and intimate (31). However, as pointed out in the findings of the study, some of the past interventions, such as the unconsented HPV vaccination program in Khwisero and forced quarantines during COVID-19, negatively impacted the trust between the CHVs and the community. In addition, the work of CHVs with the government and other non-governmental organizations affected trust because of failures by these organizations to deliver promises made to the people. CHVs were perceived as thieves and liars, leading to mistrust and violence in some cases. Present and future programs need to evaluate their impact on the trust between the CHVs and communities. Trust affects how communities receive and process health information and their subsequent behavior in adhering to interventions, including during a pandemic (32).

During the COVID-19 pandemic, misinformation complicated the preventive and responsive measures that were put in place to contain the virus. The internet, social media, and other communication channels perpetuated beliefs that fueled vaccine hesitancy, mask refusal, and utilization of medications. Many of these rumors contradicted scientific evidence and complicated the COVID-19 containment measures (33). In Kenya, as shown by our findings, there was much misinformation about the COVID-19 vaccination, leading to vaccine hesitancy. These developments had a negative impact on sexual and reproductive health. A study on drivers of vaccine hesitancy among adults in Kenya reported a lack of knowledge and misinformation as contributing factors to vaccine uptake (34). To encourage COVID-19 vaccination uptake, health providers integrated the COVID-19 vaccination with maternal health services. Although this was a novel move, it discouraged women from accessing SRH services. Health providers need proper training to ensure that as much as they want to meet targets in vaccination programs, coercion should not be used, as this promotes vaccine hesitancy.

The training offered to CHVs did not give them tools or information that would have been useful to counter the misinformation. Future training programs on pandemics should equip CHVs and health providers with tools and relevant information to counter these beliefs. In addition, research should be conducted on mechanisms to encourage vaccine uptake, especially during pandemics.

Community health volunteers reported insufficient PPEs, which could be attributed to limited global supply in the early days of the pandemic. Still, CHVs also reported unequal distribution of PPEs and other related limited resources. CHVs were also poorly facilitated when they made home visits at a period when transportation was expensive due to social distancing restrictions. These findings align with previous studies (35). For proper and efficient service delivery, CHVs needed to be well-equipped, protected, and supported to offer SRH services (36). The shortages or discriminatory distribution of PPEs exposed the CHVs to the virus. It exacerbated the community's fear of exposure to the CHVs’ presence in their homes. In light of this, Ballard and colleagues (36) have advocated for CHVs to be included in the COVID-19 PPE projections and quantification estimates. Further, given the risk posed by COVID-19 in CHV delivery of SRH services and disruptions of their everyday workflow, governments need to compensate CHVs, for example, by providing transport, airtime, and different extra work assignments as they arise during a pandemic.

According to existing literature, CHVs in Kenya played a vital role in ensuring that women received SRH services during the pandemic (37). Similarly, our findings indicate that CHVs put their lives at risk to ensure women had access to SRH services. Additionally, CHVs worked with limited resources despite taking on government health activities. Meanwhile, community members expected financial support from CHVs due to the looming economic crisis brought about by COVID-19. There is a need for the Kenyan government to provide risk allowances to CHVs during pandemics, considering the risks they endure to ensure women have access to sexual and reproductive health services.

Community health volunteers experienced psychological distress from the loss of relatives to COVID-19, being stigmatized as COVID-19 carriers, and burnout from the increased workload. These findings align with similar studies that report psychological distress experienced by healthcare workers during the COVID-19 pandemic (38). The severity of these experiences led to some CHVs quitting their voluntary jobs. Despite the consequences of psychological distress on these community health providers, they were not provided with psychosocial support. Governments that rely on CHVs to offer SRH services need to invest in mental health services for health providers, especially during pandemics. Mental health support services could be in different forms, such as improving working conditions, reducing working periods, providing access to psychosocial support services, and providing opportunities for debriefing and sharing experiences among colleagues (38).

### Strengths and limitations of the study

The strength of this study lies in the collection of data from two different sites, one urban and one rural, in Kenya. The other sites offer insight into the similar and different challenges experienced by CHVs within the country. The study also triangulated data collection methods, which contributed to its trustworthiness.

This study has limitations. It is a qualitative study, and as such, its findings cannot be generalized to other contexts. However, these findings are transferable to similar contexts within sub-Saharan Africa.

## Conclusion

This study intends to highlight the challenges CHVs face in providing SRH services and their pivotal role in responding to SRH needs during moments of global health crisis. The study offers valuable insights to ministries of health, non-governmental organizations working on SRH issues in pandemics, and different healthcare actors and stakeholders. Investment in addressing these challenges is essential as it helps CHVs execute their tasks efficiently and deliver SRH services during pandemics (39, 40). We highlight key areas that could be strengthened to allow better engagement of CHVs in their SRH work within communities during epidemics and pandemics. First, investments in providing personal protective gear and financial and human resources for CHVs' work are required to deliver services to the community effectively. Second, training of CHVs to deal with misinformation about relevant medical interventions during epidemics and pandemics. Third, psychosocial support services should be availed to CHVs during epidemics and pandemics as they impact their mental health. Finally, any interventions or programs that require CHVs should ensure that they do not negatively impact communities' trust in CHVs; the destruction of trust has negative implications on the uptake and utilization of current and future health interventions.

## Data Availability

The original contributions presented in the study are included in the article/Supplementary Material, further inquiries can be directed to the corresponding author.
